# Manufacturing Pessimism: Structural Incentives and Social Psychology’s Narrative of Human Nature

**DOI:** 10.1177/17456916261458313

**Published:** 2026-07-22

**Authors:** Jan-Erik Lönnqvist

**Affiliations:** Swedish School of Social Science, University of Helsinki

**Keywords:** structural conservatism, social psychology, pessimism, incentive structures, replication crisis, narrative, ideology, canonical studies, publication bias, open science

## Abstract

Social psychology presents itself as liberal, yet its dominant narratives—obedience, cruelty, conformity, and bias—reflect a persistently pessimistic portrayal of human nature. Here I argue that this pattern is structurally produced. A two-level framework distinguishes the content level, in which pessimistic narratives circulate, from the structural level, in which incentive regimes in academic publishing, media, and pedagogy systematically select and stabilize that content. Two mechanisms are identified. A market-selection mechanism explains why attention economies favor dramatic, morally arousing findings. An institutional-stabilization mechanism explains why pessimistic narratives prove especially durable: They participate in the constitutive logic of the “psy-complex,” in which psychological expertise gains authority by rendering human conduct legible as a problem requiring expert correction. Findings that portray ordinary people as biased, obedient, or morally fragile are apt to generate the remediation apparatus on which the field’s social legitimacy depends. Archival evidence shows that classic studies were staged and selectively framed to produce pessimistic morals. The replication crisis retroactively identified much of this content as incentive-compatible rather than epistemically robust. Reform efforts (e.g., registered reports, open data, equity-oriented partnerships) can address the market-selection side; changing the institutional-stabilization dynamic requires reflexive attention to whose interests a deficit-focused discipline serves.

Conservative policymakers and commentators recently accused U.S. universities of lacking “viewpoint diversity” (Schuessler, 2024, 2025). Although the term was left undefined, a congressional hearing focused on the scarcity of conservative faculty and students suggests an ideational reading centered on ideological diversity (Schuessler, 2024). Surveys indicate that conservatives are a minority in academia (Gross & Fosse, 2012), with estimates of roughly 10% to 15% among faculty, and only 6% among social and personality psychologists (Inbar & Lammers, 2012). This concern has a longer lineage: Tetlock (1994) argued that the liberal tilt among social scientists compromised research quality, and a much discussed 2011 Society for Personality and Social Psychology keynote address renewed the debate by calling for greater viewpoint diversity. The current article does not address ideological balance as such but uses this debate as a point of entry into a different problem: why a field that understands itself as progressive has canonized a persistently pessimistic image of human nature.

The current article takes issue not with the political leanings of individual scholars but with the structural conditions that shape what gets produced, published, and taught. The analysis focuses on the dominant introductory canon and its institutional afterlife in Anglophone social psychology rather than on all subfields or national traditions within psychology. To do so rigorously, it is necessary to distinguish three senses in which the concept of “conservatism” bears on social psychology, each operating at a different analytic level. These distinctions are not merely terminological; they map onto different causal claims and different literatures in political theory and political psychology.

“Descriptive negativity” refers to the content of dominant narratives: portrayals of people as biased, obedient, susceptible to cruelty, and morally brittle. This is an empirical claim about what the canon says but not yet a claim about why it says it or whether it is ideologically conservative in any political sense. Throughout the article, “pessimistic narratives” refers specifically to this descriptive content: portrayals of ordinary people as chronically biased, prone to obedience, susceptible to cruelty, or morally fragile in ways that require expert diagnosis and correction. The term is not used in the philosophical sense of metaphysical pessimism and does not imply that the findings in question are without merit.

“Normative conservatism” refers to the partisan ideology familiar from political science and political psychology: a disposition toward resistance to change, defense of hierarchy, and preservation of tradition and authority (Freeden, 1996; Jost et al., 2003; Robin, 2011). This is the sense in which prior debates about viewpoint diversity have framed the problem—as a scarcity of scholars with conservative political commitments. The current argument does not rest on this sense. A field can be run entirely by scholars who explicitly endorse progressive politics while its structural dynamics remain conservative in a third, distinct sense.

“Structural conservatism” refers to practices and incentive regimes—in publishing, funding, pedagogy, and media—that conserve existing hierarchies, stabilize the status quo, and reproduce current knowledge formations irrespective of how individual actors identify politically (Jost & Banaji, 1994; Jost & Hunyady, 2002). This is the operative concept in what follows. Its closest theoretical anchors are the sociology of psychologization and deficit framing. Psychologization names the process by which social and structural problems are translated into individual psychological deficits requiring expert diagnosis and management (De Vos, 2009; Furedi, 2004). Deficit framing names the complementary tendency to locate the cause of inequality or dysfunction inside persons—their cognitions, attitudes, or impulses—rather than in the institutional arrangements that surround them (Valencia, 2010). Both processes are structural in the relevant sense: They describe how institutional practices are organized, not what individual researchers consciously intend. System justification theory and social dominance research (Jost & Banaji, 1994; Pratto et al., 1994) are also compatible with the argument in that they demonstrate how hierarchy-legitimating beliefs are psychologically and socially stabilized. But their primary level of analysis is the individual’s investment in existing arrangements; the psychologization and deficit-framing literatures operate at the level of institutional practice and are therefore more directly useful for explaining how pessimistic narratives get embedded in organizational life. Both belong to a broader critical tradition—variously labeled the “psy-complex” (Parker, 2000) or “governing of the soul” (Rose, 1999)—that examines how psychological expertise constitutes subjects who govern themselves through the internalization of professional norms, generating institutional authority for a field that renders human conduct legible, measurable, and improvable.

How, specifically, does structural conservatism produce a pessimistic narrative of human nature rather than simply producing dramatic or negative content in general? The answer requires distinguishing two mechanisms that operate simultaneously and reinforce each other. The first mechanism is supported directly by evidence on publication, citation, and media selection; the second is offered as an interpretative account of why pessimistic findings prove especially reusable once selected. The first is a market-selection mechanism: Academic and media markets reward content that is novel, emotionally arousing, and morally salient, and pessimistic findings—obedience, cruelty, bias, and moral contamination—satisfy these criteria reliably. This mechanism operates at the point of initial selection and is purely functional: It would favor pessimism even if pessimism carried no ideological weight whatsoever. The second is an institutional-stabilization mechanism: Once pessimistic narratives are selected through ordinary attention dynamics, they prove unusually durable because they can be embedded in institutional scripts of supervision, correction, training, and expertise. A claim that ordinary people will comply with atrocity (Milgram), turn cruel under minimal role assignment (Zimbardo), or harbor inescapable prejudice (implicit association test; IAT) is not only dramatic but also institutionally actionable. It authorizes and sustains the apparatus of expert management, professional remediation, and organizational oversight. This is precisely the constitutive logic of the psy-complex: Psychology gains social authority by identifying problems in ordinary persons that require expert correction, and findings that portray human nature as flawed, biased, or dangerous are therefore more institutionally reusable than findings that portray it as cooperative or resilient (Parker, 2000; Rose, 1999). Prosocial findings—that people cooperate, trust, resist, and lead ethically—are equally well supported empirically (Penner et al., 2005; Rand & Nowak, 2013), but they are not equally easy to embed in training regimes, audit processes, or corrective interventions. That asymmetry in institutional reusability helps explain why pessimistic narratives remain canonical long after their evidentiary basis has been qualified or contested. The market-selection mechanism explains the initial advantage of pessimistic content; the institutional-stabilization mechanism explains why that content accumulates authority and persists once selected. Together, they explain how markets embedded in hierarchical institutions reward content that is both attention-capturing and institutionally reusable—which is precisely the intersection that pessimistic narratives about human nature reliably occupy.

The argument proceeds in two steps, each linked to one level of the framework. Level A (ideational/content) comprises pessimistic narratives—portrayals of people as biased, obedient, and morally brittle—that circulate easily across academic and media domains. Level B (structural/mechanisms) comprises incentive regimes and institutional practices that conserve the status quo and preferentially produce, select, format, and stabilize Level A content.

The rest of the article shows how Level B mechanisms select and cement Level A pessimism through both of the mechanisms identified above. First, I document that social psychology’s canonical studies align with Level A by portraying human nature in deeply pessimistic terms—and that this alignment was not accidental but produced by design choices, selective reporting, and rhetorical framing that made each finding maximally attention-capturing at the moment of selection and especially easy to embed in institutional scripts once canonized. Second, I show that the persistence of these narratives is driven by a selection environment in which prestige hierarchies, publication incentives, and media dynamics jointly reward pessimistic accounts above prosocial ones—not because anyone conspires to this end but because pessimistic narratives are more compatible with the expert-authority structures through which the field’s organizational life is organized. None of this requires individual researchers to hold conservative political commitments; it requires only that they operate within institutions in which negativity, moral arousal, and institutionally actionable conclusions are structurally rewarded.

I position the argument within critical psychology, which treats psychological knowledge as contingent on history and co-constructed with power/knowledge formations (Hook, 2007). What passes as “human nature” in the social-psychology canon is a discursive product of methodological and institutional choices—precisely the Level B incentives that format and stabilize Level A content. Pushing back requires a more reflexive methodology and institutional redesign (Burman, 2008; Parker, 2000).

## Classic Experiments and the Construction of Pessimism

### Milgram and the manufacture of obedience

Milgram’s shock experiments are read as a parable of moral collapse under authority: Ordinary citizens repeatedly press switches labeled up to 450 volts because an experimenter tells them to (Milgram, 1963, 1974). Set against the contemporaneous Eichmann trial and Hannah Arendt’s thesis of the banality of evil, the experiments quickly came to represent a disturbing claim about human nature: Under minimal institutional authority, people will comply with reprehensible demands (Milgram, 1974; Newman & Erber, 2002). The studies have merit as demonstrations of situational influence. The concern here is not that such influences are absent but that the pessimistic narrative was strategically assembled, selectively reported, and then overinterpreted as a settled verdict on human nature.

What the archives and historiography show is that the pessimistic moral was not simply discovered; it was assembled. De Vos (2009) reconstructed the timing of the initial sessions so that they coincided with the climax of the Eichmann proceedings, ensuring topical salience for an obedience narrative. Russell’s account suggested that Milgram carefully iterated the procedure to elicit high rates of compliance and then framed the program as a theoretically rich discovery after journal editors criticized early submissions as merely an atheoretical “demonstration” (De Vos, 2009; Russell, 2011). The widely used teaching film, as Millard (2014) demonstrated, was edited to highlight the most spectacular obedience sequences and to downplay variability and resistance. Rhetorical analyses of the postsession interviews indicated that participants were repeatedly pushed to account for their actions in terms of “obedience to the experimenter,” with alternative accounts pressed past until obedience was avowed (Gibson et al., 2018). Perry’s (2013, 2019) work added that many participants doubted the shocks were real, a detail largely absent from the canonical narrative. The familiar “blind-obedience” moral (Level A) was not discovered but manufactured—via procedural iteration to maximize compliance, interview steering toward an obedience account, and editorial/filmic curation that stabilized a single‑line story. Crucially, this formatting was anchored to the Holocaust frame, which secured topical salience and moral urgency for an obedience parable.

### Zimbardo and the staging of tyranny

The Stanford Prison Experiment (SPE) is routinely taught as proof that toxic roles alone can drive cruelty: Assign healthy students to “guard” and “prisoner,” remove explicit leadership, and watch sadism erupt because situations and uniforms overpower conscience (Zimbardo, 1969, 1973, 2007). The pessimistic lesson is powerful and readily disseminated. The study has been influential in highlighting situational determinants of behavior. The concern here, again, is not that situational forces are irrelevant but that the SPE’s canonical narrative was produced by staging and selective framing that went well beyond what the data warranted.

Archival reconstructions show that SPE’s cruelty was elicited from above and then retold as spontaneous. Le Texier (2019, p. 287) documented explicit coaching of guards to create “boredom, frustration, fear, arbitrariness, loss of privacy, loss of freedom of action, loss of individuality, and powerlessness,” down to operational details such as blindfolds and strip searches. That active prompting largely disappeared in the published conclusion that aggression “was emitted simply as a natural consequence of being in the uniform of a ‘guard’” (Haney et al., 1973, p. 12). Zimbardo’s (2003) own retrospective description of the study as a “Greek drama,” complete with costumes and auxiliary actors, underscores that the experiment was conceived as a staged spectacle designed for public impact. The BBC Prison Study offers a telling counterexample: When guards were not coached and explicit leader demands were absent, coercion did not take hold and participants organized along more egalitarian lines (Haslam & Reicher, 2006). Level A pessimism—that “roles inexorably turn us cruel”—was secured by construction and selection and then amplified at dissemination while introductory texts downplayed methodological critiques and demand characteristics, sustaining the “roles-make-us-cruel” storyline (Griggs, 2014).

### Robbers Cave and the orchestration of conflict

Realistic conflict theory is typically illustrated by a field demonstration in which boys at a summer camp are divided into two groups and placed in competition for scarce resources, producing intergroup hostility; cooperation is later reinstated through superordinate goals (Sherif et al., 1961). Within the canon, it is often cast as social psychology’s *Lord of the Flies* moment: evidence that competition alone generates an “us-versus-them” dynamic. The insight that resource competition can generate intergroup tension has theoretical value. What the archives show is that the specific narrative of spontaneous hostility was manufactured rather than observed.

Drawing on unpublished archives and interviews, Perry (2019) demonstrated that the hostility at Robbers Cave was orchestrated by the researchers and subsequently presented as spontaneous. The research team actively provoked hostilities—including destroying property and falsely blaming the rival group—to escalate minor frictions into antagonism. The official publications omitted these interventions, presenting hostility as a spontaneous consequence of resource competition. Perry also pointed to a suppressed precursor camp where boys in 1953 resisted attempts to be turned against each other; that failure to elicit animus received scant attention in the origin story. As Smith and Haslam (2012) noted, classic studies endure in part because they are widely taught and narratively compelling.

### Asch and the selective story of conformity

Asch’s (1951) line‑judgment experiments are the classic demonstration that individuals will publicly endorse an obviously wrong majority. Approximately 75% of participants conformed at least once, and the mean conformity rate on critical trials was about 32%—figures often cited to support a pessimistic claim about the fragility of independent judgment. The concern here is one of selective emphasis rather than fabrication: Roughly 68% of responses on critical trials were independent, and a notable minority of participants never conformed. Moreover, the artificiality of the task opens the door to informational influence and task uncertainty, not merely fear of social disapproval. Yet Asch’s studies are typically presented as a straightforward demonstration of conformity rather than as a mixed pattern that also includes substantial independence (Griggs, 2015; Smith & Haslam, 2012). The case illustrates how a pessimistic headline (“people conform”) can be emphasized over an equally possible counterlesson (“many resist”) through selective reporting and pedagogical framing.

### Tajfel and the engineered clarity of bias

Tajfel’s minimal group paradigm shows that mere categorization can elicit in-group favoritism even absent material conflict; it launched social identity theory and self‑categorization theory (Tajfel et al., 1971). There is no archival evidence of wrongdoing. The critique concerns the paradigm’s artificially high evidentiary clarity and its selective dissemination as a general claim about chronic intergroup bias. Participants are randomly categorized, prevented from using familiar allocation heuristics (merit, need, reciprocity, prior contact), and asked to decide on matrices structured to highlight between‑group contrasts. In these settings, participants typically realize they are part of an experiment and may adopt demand‑consistent strategies (Orne & Holland, 1968). The paradigm’s engineered clarity yields demonstrations that are legible and assessment‑friendly and well suited to textbooks and lectures (Smith & Haslam, 2012), but they come at the cost of ecological validity (Gozli, 2020; Smedslund, 1996). Outside the lab, allocation typically draws on richer information and is bounded by norms that curb blatant partiality. As with Asch, canonization owes more to selection pressures than to rigging, privileging simple, memorable morals about chronic bias over a more nuanced account that would include the conditions under which bias is attenuated.

### From selection to canonization: why pessimism endures

Across these exemplars, archives and testimonies document construction—prescriptive instructions, engineered conflict, and post hoc editing that distills messy behavior into a single narrative of human frailty under pressure. These choices generated not only publishable findings but also moralized takeaways—pithy lessons about human depravity—that journals, textbooks, and media routinely privilege. Crucially, the concern in each case is not wholesale collapse of the research program but the more precise pattern of selection and overinterpretation: Pessimistic framings achieved canonical status through incentive‑compatible packaging, persisting in curricula despite documented tensions with the archival record.

I examined the curriculum by analyzing chapter‑level page allocation across three widely used introductory textbooks—Hogg and Vaughan (2017), Kassin et al. (2024), and Gilovich et al. (2023). I classified conformity, stereotyping, prejudice, discrimination, and aggression as negative depictions of humanity and helping, prosociality, and cooperation as positive depictions. Page spans were calculated from a chapter’s first page to the first page of the next chapter. Across the three textbooks, negative topics received roughly four times more space than prosocial topics (77%–80% vs. 20%–23%; see Table 1), consistent with a curriculum that foregrounds pessimistic portrayals of people. The asymmetry is striking given the cumulative evidence for prosocial behavior, cooperation, and generalized trust accumulated over the same decades (for reviews, see Penner et al., 2005; Rand & Nowak, 2013). That cooperation, altruism, and trust literatures are comparatively undercanonized—despite substantial replication records—reinforces the selection argument. The disparity is not driven by differential evidentiary quality but by differential incentive compatibility—specifically by the double advantage that pessimistic narratives hold: They simultaneously capture more attention in media and citation markets and are more ideologically compatible with the hierarchy-maintaining functions of the institutions in which the discipline is embedded.

**Table 1.** Share of Textbook Pages Devoted to Negative Versus Positive Topics (Simple Chapter Page Counts)

Reference	Negative pages	Positive pages	Total pages	Negative %	Positive %	Negative % of book	Positive % of book
Gilovich et al. (2023)	130	33	163	79.8	20.2	16.9	4.3
Hogg & Vaughan (2017)	130	38	168	77.4	22.6	16.1	4.7
Kassin et al. (2024)	154	44	198	77.8	22.2	18.8	5.4

Note: Page counts were computed as the interval from a chapter’s start page to the next chapter’s start page. Conformity, stereotyping, prejudice, discrimination, and aggression were classified as negative, whereas humanity, helping, prosociality, and cooperation were classified as positive.

## Modern Variants of the Classic Template

The continued prominence of the classic studies in textbooks, scholarly framing, and public discourse underscores that their pessimistic narratives are not merely historical artifacts; they provide the template that contemporary research continues to elaborate and amplify. Social psychology can be understood in part as an intellectual response to the atrocities of World War II and the Holocaust because the classic experiments were attempts to understand how ordinary individuals could become complicit in systemic violence and genocide (Newman & Erber, 2002). As shown above, this historical origin remains visible in contemporary textbooks, virtually all of which include chapters on conformity, obedience, aggression, intergroup conflict, and antisocial behavior. Contemporary work on these themes has, in some cases, turned even darker in its narrative framing.

The IAT, developed by Greenwald et al. (1998), became the most influential tool in social psychology for measuring implicit attitudes. Using this method, researchers reported widespread pro‑White and anti‑Black preferences among White Americans, even among those who explicitly endorsed egalitarian beliefs. Popular outlets cast implicit bias as universal and framed the IAT as revealing a deep, automatic moral deficit. A BBC article entitled “Implicit Bias: Is Everyone Racist?” reported that most test takers show a pro‑White bias (BBC, 2017). *The New York Times* relayed Project Implicit’s 70% pro‑White preference figure (Tierney, 2008). *Time* magazine presented implicit bias as something “everybody” must confront (Luscombe, 2019). This rhetoric suggested diagnostic or behavioral predictive power beyond what later meta‑analyses supported: Meta‑analytic estimates indicated weak—and at best modest—relations between IAT scores and discriminatory behavior (Forscher et al., 2019; Oswald et al., 2013). The same narrative was reinforced in introductory textbooks, in which implicit bias and the IAT became staple content in chapters on attitudes and prejudice (e.g., Myers & Twenge, 2019). The gap between narrative and evidence illustrates how incentives favor and amplify pessimistic stories: Limited test–retest reliability and weak behavioral links did not prevent textbook entrenchment and sustained media uptake.

Fields and Fields (2012) articulated the concept of “racecraft” to refer to practices that create racism and reify race by treating it as a fixed psychological category rather than a social construct. One could argue that the IAT contributes to the construction of racial difference, reinforcing the very hierarchies it seeks to dismantle—a dynamic consistent with system justification processes (Jost & Banaji, 1994). The point is not that research on implicit bias lacks any merit but rather that its most dramatic claims outran its evidentiary base and that the incentive structure rewarded that overreach.

Beyond the IAT, contemporary social and moral psychology have often advanced pessimistic portrayals of humanity through the disgust-morality literature. Disgust was cast as a key driver of condemnation and dehumanization (Haidt, 2001; Rozin et al., 1999). Claims about bodily disgust shaping moral life—most famously the Macbeth effect—were widely disseminated. Mainstream outlets framed handwashing as able to “wash away guilt” or “reset” thinking (Carey, 2006; Liberatore, 2017; Nilsson, 2006). Later high‑powered replications and reanalyses reported much smaller or null effects and context‑dependent links between disgust and ideology (Donner et al., 2023; Elad-Strenger et al., 2020; Johnson et al., 2014; Landy & Goodwin, 2015; Ruisch et al., 2022). Later preregistered multilab replications of influential disgust paradigms reported attenuated or null effects, complicating the claim that incidental bodily disgust reliably shapes moral judgment (Ghelfi et al., 2020; see also Landy & Goodwin, 2015). This pattern—persistence of a pessimistic narrative despite accumulating disconfirmatory evidence—is precisely what the selection argument predicts: The dramatic framing had already been institutionalized through citations, textbooks, and media uptake before the replication evidence arrived, and the incentive structure provided no strong mechanism to dislodge it. The critique targets selective reporting, rhetorical framing, and overinterpretation that portrays a pessimistic view of human nature as more definitive than the evidence warrants.

The institutional-stabilization mechanism has documented consequences. A substantial amount of critical research now examines implicit-bias training in organizational settings, finding that these interventions individualize inequality by locating its cause inside individual minds and extend expert authority without altering the structural conditions that produce disparities (Atewologun et al., 2018; Noon, 2018). These findings are consistent with the psy-complex critique (De Vos, 2009; Furedi, 2004; Parker, 2000; Rose, 1999): Social and structural problems are translated into individual psychological deficits, generating both the diagnosis and the remediation market while extending the authority of psychological expertise into organizational life. The literature on obedience and conformity follows a parallel logic in pedagogy in that both traits are taught as revealing something dangerous about unmanaged persons, implicitly authorizing the oversight and authority structures that institutions represent. Žižek’s (2001) concept of “disavowal” is also useful here as a supplementary lens. Many researchers recognized the IAT’s psychometric limitations and weak behavioral links yet continued to deploy it in training contexts. This “acting as if” is not mere hypocrisy; it can be understood as a collective investment in a narrative whose institutional utility persists even when its evidentiary force is contested. The ideological function of the pessimistic narrative is not a hidden agenda; it is built into the reward structure of a field that gains social legitimacy precisely by identifying problems in ordinary people that expert intervention must manage (for a discussion of what psychologists professed vs. believed, see Lönnqvist, 2025).

## Replication Crisis: Structural Roots and Reform Opportunities

The replication crisis is diagnostic rather than merely technical: It exposes how incentive structures shaped what counted as knowledge. Large‑scale replication efforts show that many of social psychology’s most dramatic, counterintuitive claims were not durable regularities but fragile narratives (Cesario, 2021; Ebersole et al., 2016; Hagger et al., 2016; Klein et al., 2014; Open Science Collaboration, 2015). The point is not that nothing replicates but that the crisis retroactively identifies much of the field’s most visible content as selected content.

The replication crisis makes the incentive regimes that govern funding, coverage, and teaching visible. Attention‑driven selection rewards spectacle over stability: Studies that later fail to replicate accrue more citations than those that succeed (Serra-Garcia & Gneezy, 2021). Research on replication forecasting shows that even laypeople can predict nonreplication with above‑chance accuracy when given minimal cues about evidential strength (Dreber et al., 2015; Gordon et al., 2021; Hoogeveen et al., 2020). This finding speaks to systemic payoffs rather than to conscious editorial intent: The issue is not that journal editors deliberately publish nonreplicable results while knowing they will fail but that the structural incentives rewarding surprise, novelty, and immediate engagement make counterintuitive results a rational selection choice even when broader epistemic costs are foreseeable. The same payoff structure explains why journals have historically preferred counterintuitive results—not through awareness of nonreplicability but through an attention‑driven selection logic that systematically favors them.

Reform efforts can be understood as attempts to redesign these incentive structures. One prominent example is the registered report format, which is now used by more than 278 journals (Lin et al., 2024). Yet adoption remains limited in most fields. Among the domains associated with major replication projects, uptake is 1% or less in all but psychology, in which it has reached 7% (Lin et al., 2024). By shifting editorial commitment to the methodological plan before results are known, registered reports help reduce publication bias and selective reporting (Center for Open Science, n.d.). Preregistration, open data, and transparent workflows—supported by infrastructures such as the Open Science Framework and organizations such as Framework for Open and Reproducible Research Training (FORRT)—aim to curb undisclosed analytic flexibility and strengthen reproducibility. At the intergovernmental level, UNESCO’s *Recommendation on Open Science* explicitly links transparency, accessibility, and cooperation to both reproducibility and global equity (UNESCO, 2021).

Equity‑oriented frameworks not only advance openness but also reshape the economic incentives underlying conventional practices. Postextractivist and decolonial approaches prioritize negotiated expectations, reciprocity, and shared authorship, reducing “parachute” research dynamics and promoting evidence that is locally relevant, context-sensitive, and cumulatively useful (Godrie, 2025). Funding schemes that incorporate diversity, equity, inclusion, and accessibility criteria—such as Horizon Europe’s requirement for gender-equality plans and its gender‑dimension evaluation—explicitly link funding to inclusive processes (European Commission, 2021). These measures can broaden participation in defining research agendas and shape how findings are evaluated, reducing the structural preference for decontextualized, media‑ready narratives. Their effects are likely uneven and contingent, however, because nominal compliance and persistent parachute practices remain in the absence of enforcement and cultural change (Dutta et al., 2023; Gewin, 2023).

These reforms advance critical psychology’s long‑standing emphasis on reflexivity in knowledge production and the redistribution of epistemic authority (Parker, 2000). In Foucauldian terms, they transform the apparatus that organizes psychological truths, reshaping the processes by which claims are structured, disseminated, and taught (Hook, 2007). Pedagogical changes further resist developmentalist assumptions that naturalize deficit and disorder, positioning classic exemplars as historically situated selections rather than reflections of inherent essence (Burman, 2008). At the same time, a similar pattern of structural conservatism is evident in many widely promoted “solutions,” which often recast structural problems as individual deficits. Initiatives such as sustainability nudges, bias‑reduction workshops, and mindfulness programs in schools are frequently presented as progressive but commonly reframe structural problems as individual shortcomings while institutional arrangements remain unchanged (Burnette et al., 2023; Gaffney et al., 2021; Kuyken et al., 2022).

## Structural Drivers of Negativity and Moral Drama

This section shifts from disciplinary incentives to the media and academic environments that select and disseminate research claims. The following subsections trace four interconnected mechanisms: prestige hierarchies and cumulative advantage; novelty bias in editorial selection; article processing charges (APCs)/paywall barriers and Western, educated, industrialized, rich, and democratic (WEIRD) samples; and media selection dynamics. Together, these mechanisms form a selection environment in which pessimistic, dramatic, and morally salient narratives are systematically advantaged over reproducible, prosocial, or contextually nuanced accounts. Crucially, these mechanisms do not simply favor dramatic content in general: Pessimistic findings occupy a privileged intersection, satisfying both the attention criteria that novelty bias selects for and the institutional-utility criteria that make them specifically reusable in training, oversight, and remediation contexts.

### Prestige hierarchies, cumulative advantage, and novelty bias

Academic publishing operates through prestige hierarchies in which a small number of flagship journals exercise disproportionate control over visibility and career capital. Publication in high-impact outlets generates cumulative advantage: Cited work attracts further citations, authors at prestigious institutions are more likely to be accepted at prestigious outlets, and institutional prestige predicts where scholars are hired and promoted (Clauset et al., 2015; Wapman et al., 2022). This cumulative dynamic concentrates epistemic authority and entrenches the narrative preferences of established research programs.

Editorial incentives for novelty compound these dynamics. Across 49 journals, more novel submissions were more likely to be accepted, and editors selected strongly for novelty even after reviewer recommendations were taken into account (Teplitskiy et al., 2022). Similarly, reviewers at high-prestige outlets show preference for counterintuitive findings (Muradchanian et al., 2023). Smaldino and McElreath’s (2016) evolutionary model extended the point from individual decisions to the structure of the system, showing that competition tends to favor lower statistical power and higher false discovery rates when career rewards track output more than reliability. The combined effect of prestige hierarchies, cumulative advantage, and novelty-biased editorial selection is to create a structural preference for the kind of striking, pessimism-coded findings that made the canonical studies famous.

### APC barriers, WEIRD sampling, and the reproduction of hierarchy

APCs in open‑access publishing, often exceeding $2,000 to $5,000 per article, concentrate publishing capacity among well‑funded laboratories in wealthy institutions and countries. Researchers without institutional APC subsidies—disproportionately those in the Global South and at teaching‑focused institutions—face barriers to publishing in high‑visibility open‑access venues. Combined with paywalled subscription journals that restrict readership to institutions with library budgets, these financial structures reproduce the hierarchy in knowledge production: whose research gets published, who can read it, and whose populations are studied.

WEIRD sampling compounds these dynamics. When research populations are systematically drawn from psychology undergraduate pools at a handful of elite universities in North America and Western Europe, the findings generalized as universal claims about human nature may in fact reflect culturally specific patterns. This matters for the pessimism argument: Cooperative, prosocial, and trust-based behaviors show substantial cross‑cultural variation (Henrich et al., 2010), and the specific ecological conditions of WEIRD samples may amplify the salience of competitive, individualistic, and conformist responses. The structural conservatism of WEIRD sampling thus operates by restricting the empirical base from which claims about “human nature” are drawn, systematically underrepresenting the populations and contexts in which more optimistic accounts might have stronger evidentiary support.

### Media selection dynamics

Legacy newsrooms have codified “what sells” through enduring news values and formats that systematically favor drama, negativity, and visual intensity. Gatekeeping research has long established that events marked by pronounced negativity, significant magnitude, and unexpectedness systematically rise in the running order and occupy front‑page positions (Galtung & Ruge, 1965; Harcup & O’Neill, 2001, 2017). In broadcast news, producers consistently lead with crime and violent events because these stories stimulate audience engagement (Hamilton, 1998, 2003). Viewers exhibit stronger physiological responses to negative news than to positive news, a cross‑national pattern that helps explain why such frames sustain attention (Soroka et al., 2019).

Although the commercial preference for negative content predates the social platform era, algorithmic ranking and recommender systems now reproduce and often intensify this logic online. Negativity demonstrably drives engagement: Randomized headline A/B tests show that adding negative words significantly increases click‑through, regardless of story content (Robertson et al., 2023). The circulation of a story accelerates when moral‑emotional language is present—anger, disgust, and outrage strongly drive sharing within ideological networks (Brady et al., 2017). Content that is novel or false—often infused with fear or disgust—spreads more rapidly and widely than truthful content (Vosoughi et al., 2018). Users share negative articles nearly twice as often as positive ones, with the strongest amplification occurring in political news targeting out-groups (Watson et al., 2024). Recent work shows a robust negativity bias in online news consumption and sharing, complementing earlier evidence that negative language increases click-though and that negative articles are more likely to be shared on social media (Robertson et al., 2023; Watson et al., 2024: Zhang et al., 2024).

The university press office, frequently acting as a link between journals and mass media, can introduce exaggeration prior to any involvement by reporters. University and journal press releases frequently present correlational findings as causal, and subsequent news coverage typically reflects the language of the release (Sumner et al., 2014, 2016). Institutional releases, designed to attract attention, often emphasize risk, urgency, and moral stakes, contributing to the wider diffusion of pessimistic framings through headlines and social media (Bratton et al., 2019; Robertson et al., 2023; Watson et al., 2024).

### The selection environment as a whole

Taken together, these mechanisms create a selection environment. Prestige hierarchies concentrate epistemic authority and entrench narrative preferences through cumulative advantage. Novelty bias in editorial selection rewards striking, counterintuitive claims over reproducible ones. APC barriers and WEIRD sampling restrict both who can publish and whose populations are counted as evidence of human nature. Media selection dynamics then amplify whatever pessimistic, morally arousing content the academic system produces, generating further citation incentives through visibility. Negativity and moral arousal generate attention; attention converts into prestige; prestige influences what is taught, cited, and assigned. None of these processes require ideological self‑identification among researchers. They require only that researchers function within markets in which negativity, moral arousal, and surprise are rewarded. The prosocial literature—cooperation, altruism, generalized trust, and resistance—is not absent, but it is comparatively undercanonized because it is comparatively less incentive‑compatible with this selection environment.

A political‑economy lens helps explain why these incentives persist and why they favor pessimism specifically. Academic psychology is embedded in markets that determine visibility and career capital and in universities that prioritize branding, competitiveness, and revenue (Newfield, 2016; Slaughter & Rhoades, 2004). Publication and evaluation are controlled by an oligopoly of publishers and entrenched journal hierarchies that dictate circulation and prestige (Fanelli, 2012; Larivière et al., 2015). In this environment, the market-selection mechanism and the institutional-stabilization mechanism described in the introduction converge. Following Marx (1859/1970), ideology is understood as rooted in conditions of production rather than in individual belief states: What gets produced is not simply what researchers choose to study but what the conditions of production make rewarding to study. A field embedded in capitalist media logics and prestige hierarchies will tend to produce, circulate, and canonize claims that are simultaneously attention-capturing and compatible with the institutional authority structures on which the field’s own legitimacy depends. Pessimistic narratives of human nature are precisely such claims: They are dramatic enough to attract media and citation attention, and they carry the implicit ideological warrant that existing structures of authority, correction, and oversight are necessary. In contexts in which attention, novelty, and hierarchy/compatibility are jointly incentivized, the dominance of pessimism‑coded narratives is structurally overdetermined.

By calling social psychology “structurally conservative,” I am not imputing partisan leanings to its practitioners. The argument is that two mechanisms together help explain why this particular field has canonized this particular kind of pessimistic content. The market-selection mechanism explains why dramatic, emotionally arousing, and counterintuitive claims circulate more widely than careful, cumulative, or prosocially framed ones: This is a generic feature of attention economies and prestige hierarchies and is not peculiar to psychology. The institutional-stabilization mechanism offers an interpretation of why, within the broader universe of dramatic claims, pessimistic narratives about human nature prove especially durable. The argument is interpretive rather than directly demonstrated: The claim is that pessimistic findings are more readily embedded in training regimes, professional tools, and expert-authority structures than prosocial findings are. The clearest documented case is the organizational use of implicit-bias research: Interventions based on pessimistic accounts of automatic prejudice have been widely institutionalized in diversity training programs, even as their evidentiary basis has been substantially qualified (Atewologun et al., 2018; Noon, 2018). This case is illustrative rather than exhaustive, but it displays the mechanism: A pessimistic narrative about ordinary cognition generates an expert-remediation industry that persists independently of the underlying evidentiary debate. Neither mechanism requires conscious ideological intent. Together they constitute structural conservatism as the article defines it—a system in which the conditions of production and dissemination reward content that is simultaneously attention-capturing and institutionally reusable and in which pessimistic portrayals of human nature happen to be both.

## Reconfiguring Incentives, Reimagining Narratives

At the outset, I introduced a two‑level framework. Level A refers to social psychology’s dominant narratives about human nature: pessimistic portrayals of people as biased, obedient, and susceptible to cruelty. Level B refers to the structural incentives—prestige hierarchies, cumulative advantage, novelty-biased editorial selection, APC/paywall barriers, WEIRD sampling, and media selection dynamics—that select and stabilize those narratives. In the preceding sections I have argued that the relationship between these levels is structured and directional: Level B mechanisms systematically favor Level A content. This final section explores what would change if the incentive structure were redesigned.

Registered reports represent the most direct structural lever currently available. By moving editorial commitment to the methodological plan before results are known, they decouple publication probability from result direction, substantially weakening publication bias (Center for Open Science, n.d.; Lin et al., 2024). The use of the format has increased, particularly in psychology, in which uptake has been stronger than in most other fields, and early evidence suggests that registered reports produce a higher rate of null results and are less likely to overstate effect sizes (Scheel et al., 2021). FORRT supports the integration of open science practices into pedagogy, directly targeting the educational reproduction of fragile narratives. Preregistration, open data, and transparent workflows reduce undisclosed analytic flexibility and create accountability mechanisms that make the conditions of knowledge production legible (Center for Open Science, 2024). These reforms are not peripheral; they are attempts to alter precisely the Level B mechanisms described above.

The key question is whether these reforms can overcome the structural inertia created by prestige hierarchies and cumulative advantage. There are reasons for cautious optimism. When incentives change, content changes too: The growing share of findings of null and small effect sizes in preregistered research suggests that transparency requirements can shift the distribution of published results. Funding and partnership arrangements can function as additional structural levers. Policies that require open data, equitable collaborations, gender‑equality plans, and shared data governance alter the conditions under which research is organized and rewarded, broadening participation in agenda-setting and reducing the concentration of epistemic authority in a small number of elite institutions (European Commission, 2021; Godrie, 2025).

Pedagogically, the two‑level framework offers a resource for teaching the history of the field without either uncritically transmitting its pessimistic narratives or dismissing its genuine insights. Students should learn not only the findings but also the conditions of their production, selection, and communication: the design decisions, instruction protocols, and dissemination choices that structured the presentation of canonical results. Demonstrating ongoing reforms that strengthen transparency and rigor is essential for countering undue skepticism: Showing students that the discipline is capable of self‑correction is as important as documenting its structural problems. Classic exemplars can be repositioned as historically situated selections—artifacts of a particular incentive environment—rather than as settled verdicts on human nature.

The discipline’s prevailing pessimistic view of human nature is not fixed but historically contingent. If Level B mechanisms are redesigned—if prestige hierarchies are flattened, cumulative advantage is attenuated, editorial incentives are decoupled from novelty and drama, financial barriers to publishing are reduced, and sampling practices become more globally representative—then the selection environment that has favored pessimism‑coded narratives will be weakened. Over time, this could allow cooperation, deliberation, resistance, and ethical leadership to achieve canonical status on equal footing with the long‑standing emphasis on bias, cruelty, obedience, and susceptibility to tyranny. That outcome would not require researchers to abandon critical perspectives or to adopt naive optimism about human nature; it would require only that the incentive structure stop stacking the deck against it. It bears noting, finally, that the two mechanisms identified in this article have different implications for reform. Methodological transparency—registered reports, preregistration, open data—directly addresses the market-selection mechanism by weakening the incentive to produce dramatic, unreplicable findings. The institutional-stabilization mechanism is harder to address by methodological means alone because it operates through the organizational uptake of findings rather than through their original production. Curricular reform, pedagogical reflexivity, and critical examination of how psychological findings are operationalized in training and policy contexts are the corresponding levers on that side.

Last, the structural conservatism documented in this article is not an accident of incentives alone. It reflects a constitutive feature of psychological expertise as such: The discipline gains institutional authority by rendering human conduct legible as a problem—something to be diagnosed, measured, and corrected. The concepts of “psychologization” and “deficit framing” name this dynamic precisely. When ordinary people are portrayed as biased, obedient, or morally fragile, the findings do not merely circulate; they generate the remediation apparatus that extends the field’s social legitimacy. This is why pessimistic narratives about human nature prove more durable than their evidentiary basis alone would predict: They are not simply dramatic stories but institutionally useful ones in a field whose authority depends on identifying what is wrong with people and offering to fix it. Changing what social psychology says about human nature therefore requires more than methodological reform. It requires reflexive attention to the question of whose interests are served by a discipline that systematically finds pathology in individuals while leaving structural conditions intact (De Vos, 2009; Parker, 2000; Rose, 1999).

## Transparency

*Action Editor:* Arturo E. Hernandez

*Editor:* Arturo E. Hernandez
